# Coronary Subclavian Steal Syndrome: An Unusual Cause of Angina in a Post-CABG Patient

**DOI:** 10.1155/2014/769273

**Published:** 2014-04-29

**Authors:** Usman Younus, Brandon Abbott, Deepika Narasimha, Brian J. Page

**Affiliations:** ^1^Department of Medicine, State University of New York at Buffalo, Buffalo, NY, USA; ^2^Cardiovascular Division, Clinical & Translational Research Center (CTRC), University at Buffalo, Suite 7030, 875 Ellicott Street, Buffalo, NY 14203, USA

## Abstract

Coronary subclavian steal syndrome is a rare complication of coronary artery bypass grafting surgery (CABG) when a left internal mammary artery (LIMA) graft is utilized. This syndrome is characterized by retrograde flow from the LIMA to the left subclavian artery (SA) when a proximal left SA stenosis is present. We describe a unique case of an elderly male who underwent CABG 6 years ago who presented with prolonged chest pain, mildly elevated troponins, and unequal pulses in his arms. A CTA of the chest demonstrated a severely calcified occluded proximal left SA jeopardizing his LIMA graft. Subclavian angiography was performed with an attempt to revascularize the patient's occluded left SA which was unsuccessful. We referred the patient for nuclear stress testing which demonstrated a moderate size area of anterior ischemia on imaging; the patient exercised to a fair exercise capacity of 7 METS with no chest pain and no ECG changes. Subsequent coronary angiography showed severe native three-vessel coronary artery disease with intermittent retrograde blood flow from the LIMA to the left SA distal to the occlusion, jeopardizing perfusion to the left anterior descending (LAD) coronary artery distribution. He declined further options for revascularization and was discharged with medical management.

## 1. Introduction

Coronary subclavian steal syndrome is a rare but well-recognized complication of coronary artery bypass grafting (CABG) surgery when a left internal mammary artery (LIMA) graft is used. This syndrome is characterized by retrograde blood flow from the LIMA to the distal subclavian artery (SA) to perfuse the upper extremity when a severe proximal SA stenosis is present. As a result, a coronary steal phenomenon may develop whereby the myocardium perfused by the LIMA graft can become ischemic despite the patency of the grafted vessels.

## 2. Case

We describe the case of an 84-year-old male with history of diabetes mellitus, hypertension, atherosclerotic carotid disease, and three-vessel coronary artery disease (CAD) with CABG 6 years ago, who presented with moderate intensity 5 out of 10 prolonged left-sided chest pain, worse with physical activity involving the left upper extremity. The pain persisted overnight and the patient presented the following day to the emergency department. Since bypass surgery, the patient had been doing well with no anginal symptoms and was maintained on aspirin 81 mg daily, clopidogrel 75 mg daily, metoprolol 25 mg twice daily, enalapril 10 mg daily, and metformin 500 mg twice daily. He received an LIMA graft to the left anterior descending artery (LAD) and saphenous vein grafts to the posterior descending and marginal arteries with his CABG surgery.

**Figure 1:**
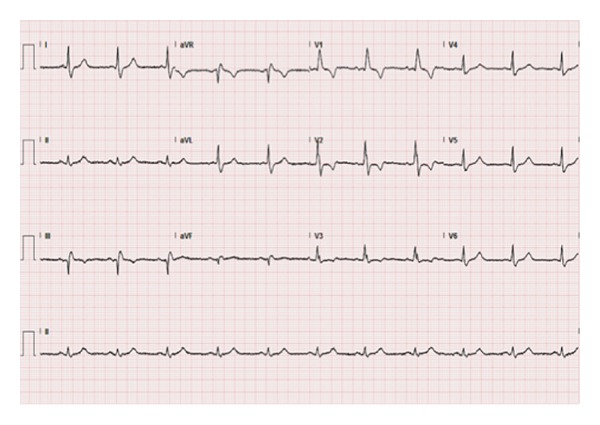
12-lead electrocardiogram showing sinus rhythm with right bundle branch block.

On physical examination the patient had an interarm blood pressure difference of 50 mm Hg (84/56 in the left arm and 135/86 in right arm), faint pulses in the left upper extremity, and equal pulses in the lower extremities bilaterally. There was no supraclavicular bruit. Electrocardiogram (EKG) showed sinus rhythm with a right bundle branch block with no new ST or T wave changes (Figure 1). Troponin I was increased to 0.09 ng/mL (normal range 0.00–0.06 ng/mL) on admission. The patient was diagnosed with a non-ST elevation myocardial infarction, was admitted to the telemetry unit, and was anticoagulated with unfractionated heparin per acute coronary syndrome (ACS) protocol.

**Figure 2:**
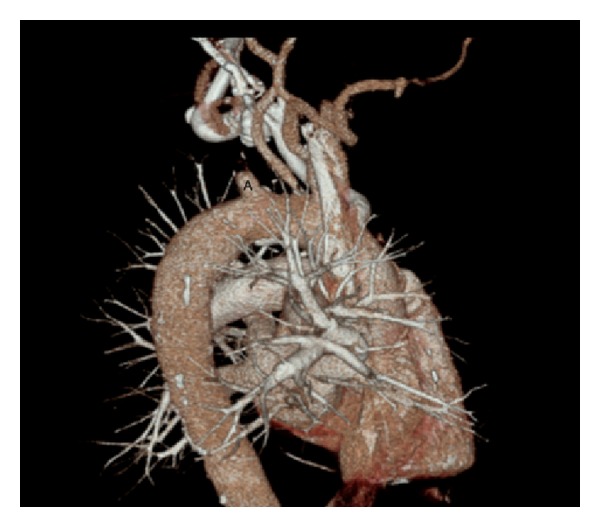
CTA of the chest with complete occlusion of left subclavian artery (A).

Given the patient's symptoms of chest pain and the significant blood pressure difference between his arms, we requested a computed tomography angiogram (CTA) of the chest to rule out aortic dissection. The study did not show evidence of aortic dissection; however, the patient was incidentally noted to have a proximally occluded left subclavian artery (SA) (Figure 2). The concern was that this could be jeopardizing his LIMA graft, and a vascular surgery consultation was requested. In the meantime an echocardiogram was performed which showed a normal left ventricular ejection fraction greater than 55% and mild concentric left ventricular hypertrophy with no significant valvular abnormalities.

**Figure 3:**
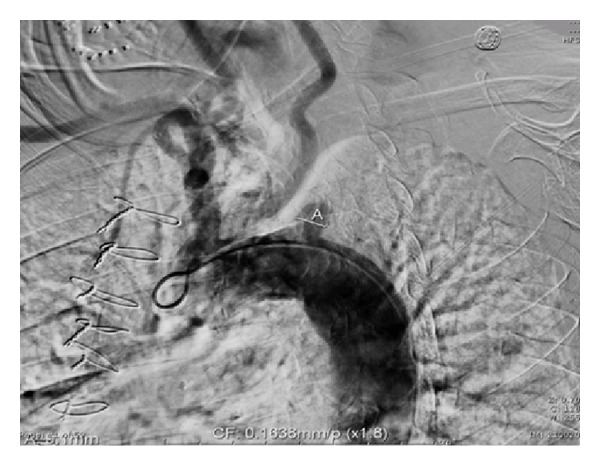
Thoracic aortogram with supra-aortic vessels. (A) Complete SA occlusion.

**Figure 4:**
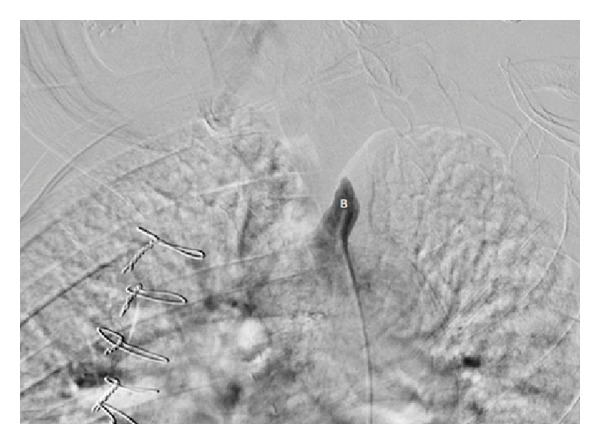
Selective subclavian angiogram. (B) Inability of the guide wire to pass through the completely occluded subclavian artery.

**Figure 5:**
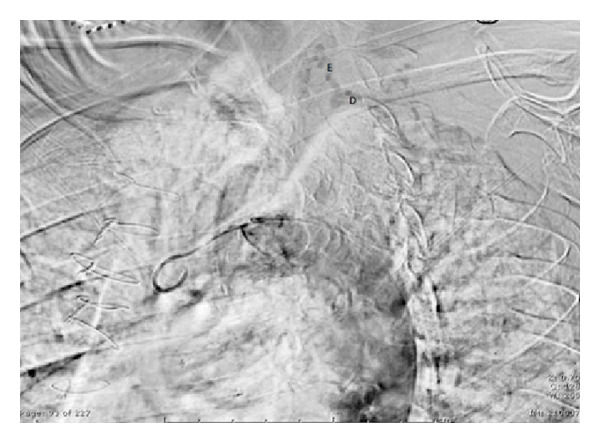
Perfusion of distal left SA (D) by retrograde filling from vertebral artery (E).

At the recommendation of the vascular surgery consultant, subclavian angiography was performed with a view towards percutaneous intervention. This confirmed complete occlusion of left SA with severe calcification just distal to its origin (Figures 3 and 4) and reconstitution of the SA at the level of the vertebral artery through collaterals from the carotid artery to the vertebral artery. The Distal SA was perfused by retrograde blood flow from the vertebral artery (Figure 5). Attempts to pass the guide wire across the lesion were unsuccessful (Figure 4), and the left SA could not be percutaneously revascularized.

**Figure 6:**
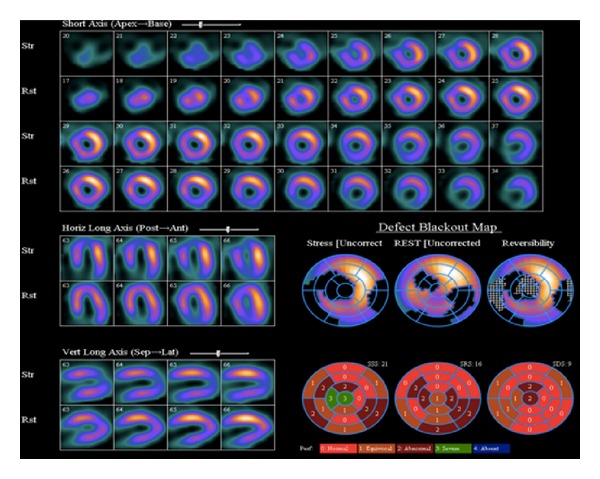
Myocardial perfusion imaging demonstrating a moderate size area of ischemia in the mid to apical anteroseptal, apical, and periapical segments.

We subsequently referred the patient for stress testing with myocardial perfusion imaging (MPI) for further risk stratification and to determine how large and severe an area of ischemia may be present from compromise of his LIMA graft. The patient exercised to 84% of his age predicted maximum heart rate and an exercise capacity of 7 METS on a standard Bruce treadmill protocol with no ECG changes, no chest pain, no arrhythmias, and normal blood pressure response to exercise. MPI showed a moderate size area of ischemia involving the mid to apical anteroseptal, apical, and periapical segments with a normal left ventricular ejection fraction of 63% (Figure 6).

**Figure 7:**
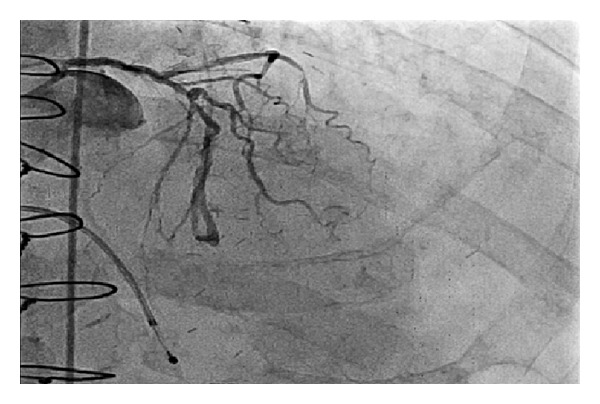
Left anterior oblique projection showing left coronary circulation with severe disease.

**Figure 8:**
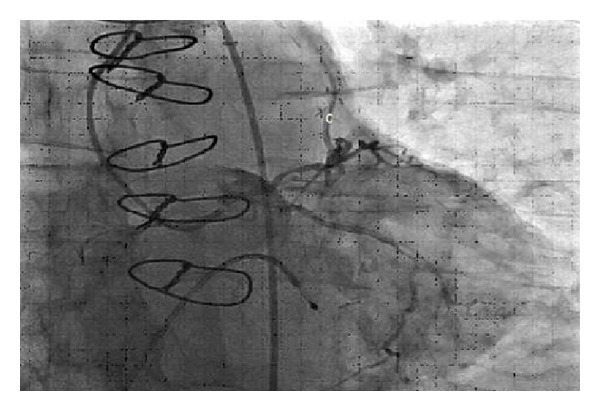
LIMA to LAD graft with retrograde blood flow (C).

Following this result, we referred the patient for left heart catheterization to assess the extent and severity of his native coronary artery disease and to determine the feasibility of percutaneous coronary intervention (PCI) to his native LAD. Coronary angiography confirmed the patency of his bypass grafts as well as severe and diffuse native CAD with 80% left main stenosis and 80–90% proximal LAD stenosis (Figure 7); there was also evidence of intermittent retrograde filling of the LIMA from the LAD into the left SA distal to the occlusion (Figure 8).

At this point other treatment options were considered including axilloaxillary, carotid-subclavian, and subclavian-subclavian artery bypasses and PCI to the left main and LAD; however, the patient declined further intervention due to the risks involved. We discontinued his ACE inhibitor as its vasodilator effects could possibly worsen the retrograde flow in the LIMA graft. The patient was advised not to participate in vigorous physical activity, especially that which would involve increased left upper extremity exertion which could worsen coronary steal. Close outpatient followup was advised. He was otherwise maintained on dual antiplatelet therapy with aspirin and clopidogrel, beta-blocker, and statin therapy.

## 3. Discussion

We describe a rare cause of chest pain in a post-CABG patient where myocardial ischemia was secondary to proximal left SA occlusion jeopardizing the LIMA graft with development of coronary steal syndrome due to retrograde blood flow from the LIMA to the distal left SA. While the use of a LIMA graft with CABG has become widely accepted as the standard of care [1], left SA stenosis can compromise anterograde blood flow in the LIMA and if severe, can result in coronary subclavian steal syndrome (CSSS). The first case of CSSS was described in 1974 [2]. Although the most common cause of angina in a post-CABG patient is progression of native vessel coronary atherosclerosis and disease in the grafts, this syndrome should also be considered in the differential diagnosis of post-CABG angina [3].

The most common cause of CSSS is ipsilateral subclavian artery stenosis caused by atherosclerosis; however, other pathological etiologies including Takayasu arteritis [4], radiation arteritis [5], and hemodialysis AV fistula [6] have also been implicated in the development of CSSS. The described time frame for the start of CSSS symptoms has ranged from 2 to 31 years after CABG, and earlier symptoms of CSSS could represent missed SA stenosis at the time of surgery [7]. The incidence of SA stenosis depends on the patient population under study. The highest incidence of SA disease is in those with established peripheral arterial disease in other peripheral arteries, which was 11.8% in one series [8]. Subclavian artery calcification has been associated with risk factors such as advanced age, hypertension, diabetes mellitus, smoking, and other nonsubclavian vascular calcification [8, 9].

A history of peripheral vascular disease along with the presence of an interarm blood pressure difference greater than 20 mm Hg has been proposed as clinical predictor of subclavian artery stenosis [10]. The presence of a supraclavicular bruit and symptoms of vertebrobasilar insufficiency including dizziness, syncope, ataxia, blurry vision, drop attacks, upper extremity claudication, and numbness are also useful clues in diagnosing the condition.

The sensitivity and specificity of an interarm blood pressure difference in predicting proximal subclavian artery stenosis were previously evaluated in 492 patients undergoing cardiac catheterization [8]. The study showed an interarm blood pressure difference of >10 mm Hg and >20 mm Hg had good specificity (85% and 94%, respectively) but low sensitivity for subclavian artery stenosis. This was in part due to the presence of multivessel disease that could affect blood pressure in both arms as 31% of patients with brachiocephalic disease were found to have multivessel stenosis [11, 12].

Noninvasive diagnostic modalities to establish CSSS include duplex ultrasonography of the supra-aortic vessels, computed tomography scanning, and magnetic resonance angiography. The presence of flow reversal, including complete retrograde flow in the vertebral artery, is a highly sensitive indicator of ipsilateral SA stenosis. Definitive diagnosis is established with direct subclavian angiography [13]. The routine performance of subclavian angiography prior to consideration of coronary bypass surgery is controversial; however, many facilities routinely screen for SA stenosis at the time of coronary angiography and include cerebral angiography and arch aortography if significant SA disease is found [13]. CSSS can be avoided using only vein conduits or with the use of free IMA or radial artery conduits. However, using all venous conduits is associated with an increase in the incidence of adverse cardiac events [1, 8].

Current treatment options for CSSS include endovascular or surgical revascularization. The endovascular approach with percutaneous transluminal angioplasty (PTA) and peripheral stenting has been considered first line treatment for SA stenosis [14–16] and has many advantages including a minimally invasive approach, shorter hospital stay, less morbidity, and avoidance of general anesthesia [14, 17] compared to surgical bypass techniques. A few drawbacks include unsuccessful recanalization of the artery as in our patient [18] and increased frequency of repeat procedures due to restenosis [19]. The incidence of restenosis was reported as 12% over a mean followup of 5.8 years [17] but has been shown to be as high as 28.5% over a follow-up period of 5 years [20]. Also continuous subclavian coronary steal when compared to intermittent steal predicts greater risk of restenosis after PTA [20].

The success rate of recanalization of an SA occlusion is significantly lower when compared with SA stenosis without occlusion [14, 17, 18]. Initial success rates of PTA/stenting for SA stenosis have been 100% in multiple studies compared to more variable outcomes with SA occlusions, with a maximum success rate of 82.1% and minimum of 47% [14, 18]. Failed PTA was highest in patients with severe calcification, as in our patient [18]. Importantly, an increased trend towards mortality (21.7% versus 50%) was observed in patients with failed revascularization [18].

Surgical methods to manage SA occlusion include carotid-subclavian, carotid axillary, axilloaxillary, and aorta-subclavian bypasses, as well as transposition of the IMA [12, 13, 21]. These surgical procedures are relatively high risk but may be the only option for revascularization in patients with a completely occluded SA. Surgical bypass has been the preferred option in circumstances where length of the lesion is > than 5 cm, where there is severe calcification and complete occlusion near the ostium of the vertebral artery [18], and where concomitant brachiocephalic and coronary artery disease is present [12, 13]. Multiple studies have shown a 10-year patency rate of surgical grafts to be more than 90% [11, 13, 21]. Carotid-subclavian bypass alone has been shown to have 10-year primary and secondary patency rates of 92% and 95%, respectively, with symptom-free survival rate of 82% at 5 years and mortality rate of 0% at 30 days. However, the mean age of patients in the study was 62 with range of 42–75. Less data is available on treatment outcomes with surgical routes in those at age greater than 80 years.

There is limited data on outcomes with medical management. In one case report, a patient who refused to have revascularization of a hemodynamically significant SA occlusion died 12 months after diagnosis. Patients with asymptomatic restenosis following revascularization have been treated with exercise rehabilitation and drug therapy [18]. Close followup of such patients is of utmost importance, as any future recurrent symptoms could be fatal due to the underlying functionally impaired conduit.
